# Inpatient Use of Metformin and Acarbose Is Associated with Reduced Mortality of COVID-19 Patients with Type 2 Diabetes Mellitus

**DOI:** 10.21203/rs.3.rs-287308/v1

**Published:** 2021-03-26

**Authors:** Willis Li, Jinghong Li, Qi Wei, Karen McCowen, Wei Xiong, Jiao Liu, Wenlijun Jiang, Robert Thomas, Mark Hepokoski, Ming He, John Shyy, Atul Malhotra, Nian Xiong

**Affiliations:** University of California, San Diego; University of California, San Diego; Wuhan Red Cross Hospital; University of California, San Diego; Wuhan Red Cross Hospital; Wuhan Red Cross Hospital; Wuhan Red Cross Hospital; University of California, San Diego; University of California, San Diego; University of California, San Diego; University of California, San Diego; University of California, San Diego; Wuhan Red Cross Hospital

**Keywords:** Coronavirus disease 2019 (COVID-19), Type 2 diabetes mellitus (T2DM), metformin, acarbose, insulin, sulfonylureas

## Abstract

Type 2 diabetes mellitus (T2DM) is a strong risk factor for complications of coronavirus disease 2019 (COVID-19). The effect of T2DM medications on COVID-19 outcomes remains unclear. In a retrospective analysis of a cohort of 131 patients with T2DM hospitalized for COVID-19 in Wuhan, we have previously found that metformin use prior to hospitalization is associated with reduced mortality. Here we continue to investigate the effects of inpatient use of T2DM medications, including metformin, acarbose, insulin, and sulfonylureas, on the mortality of COVID-19 patients with T2DM during hospitalization. We found that patients using metformin and acarbose, alone or both together, after admission were significantly more likely to survive than those who did not use either metformin or acarbose. Thus, our analyses suggest that inpatient use of metformin and acarbose together or alone during hospitalization should be studied in randomized trials.

## Introduction

COVID-19 has had devastating consequences for many patients globally. At present remdesivir and dexamethasone are the only proven therapies ^1, 2^; thus, therapeutic advances are still required. A number of risk factors for COVID-19 have been reported including COPD, diabetes, obesity, advanced age, hypertension and other factors ^3–5^. The elucidation of mechanisms underlying these risk factors may have benefits for stratification of high risk patients and may help to guide pharmacological targets for afflicted patients.

Regarding diabetes, the optimal therapy from the perspective of COVID-19 is unclear. We have recently reported an association between pre-hospitalization metformin use and improved risk of mortality, leading us to consider the impact of inpatient pharmacotherapy ^6^. Inpatient therapy for glucose control in the US typically involves discontinuation of oral agents upon admission and initiation of insulin therapy ^7^.

However, in some countries, including China, oral agents are used in some cases preferentially even in the inpatient setting. Typically, oral agents are continued after hospitalization and the doses are adjusted according to guidelines, similar to the outpatient settings^8^. We and others have previously reported on the risks and benefits of inpatient glycemic control ^9, 10^; however, the best strategy to apply to COVID-19 patients remains unclear. In theory insulin may be beneficial as it could achieve glucose control effectively and avoid hyperglycemia-related complications such as immunosuppression and oxidative stress. On the other hand, some oral agents such as metformin may activate the AMP-activated protein kinase (AMPK), which has important effects on autophagy ^11^. Acarbose is an alpha-glucosidase inhibitor, which serves to delay glucose absorption and thus reduce the post-prandial insulin spike ^12^. Acarbose has been shown to have potentially important effects on the gut microbiome although the impact of these changes is unclear ^13^. Acarbose is a commonly used diabetes medication in China for post-prandial glucose control due to the relatively high-carbohydrate diets.

Based on this conceptual framework, we sought to test the hypothesis that oral agents would have differential effects on COVID-19 inpatients.

Specifically, we tested whether metformin and acarbose use were associated with improved outcomes as compared to insulin therapy in COVID-19 inpatients in China. Based on the pace of the pandemic, we were unable to perform a randomized trial, but rather conducted an observational study to help inform subsequent research.

## Methods

### Study Population and Procedures

131 patients with COVID-19 pneumonia and T2DM hospitalized in Wuhan Red Cross Hospital (WRCH) in Wuhan, China, from January 23, 2020 to March 19, 2020 were included. The same patient cohort has been previously reported for pre-hospital medication use^6^. COVID-19 was diagnosed using reverse transcription polymerase chain reaction to test for SARS-CoV-2 genes from nasopharyngeal swab samples, according to the World Health Organization (WHO) interim guidance ^14^. The diagnosis of T2DM was determined using clinical records according to the WHO diagnostic criteria for T2DM ^15^.

The study protocol was approved by the WRCH Ethics Committee and written informed consent was obtained from the patients included in the study.

Clinical and outcome data were reviewed and collected from electronic medical records by a trained team of physicians. The data from the medical records included demographic information, medical history, clinical characteristics, laboratory results, treatments, duration of hospital stay, and outcomes. Our main outcome was mortality among patients with COVID-19 and T2DM. Patient information was de-identified for privacy and confidentiality. Two independent researchers reviewed the database for accuracy.

### Statistical Analysis

Chi-squared test and Fisher’s exact test were used to compare univariate differences between survivors and non-survivors. Univariate logistic regression models were used to analyze effects of continuous variables on mortality. Multivariate logistic regression models were also used to assess simultaneous effects of continuous, binomial and categorical variables on survivability or mortality. Statistical significance was set at α (*p*-value) of less than 0.05. Statistical analyses were performed using the R programming language or MATLAB ®.

## Results

### Diabetes medications for COVID-19 with T2DM before and during hospitalization

**Figure 1:**
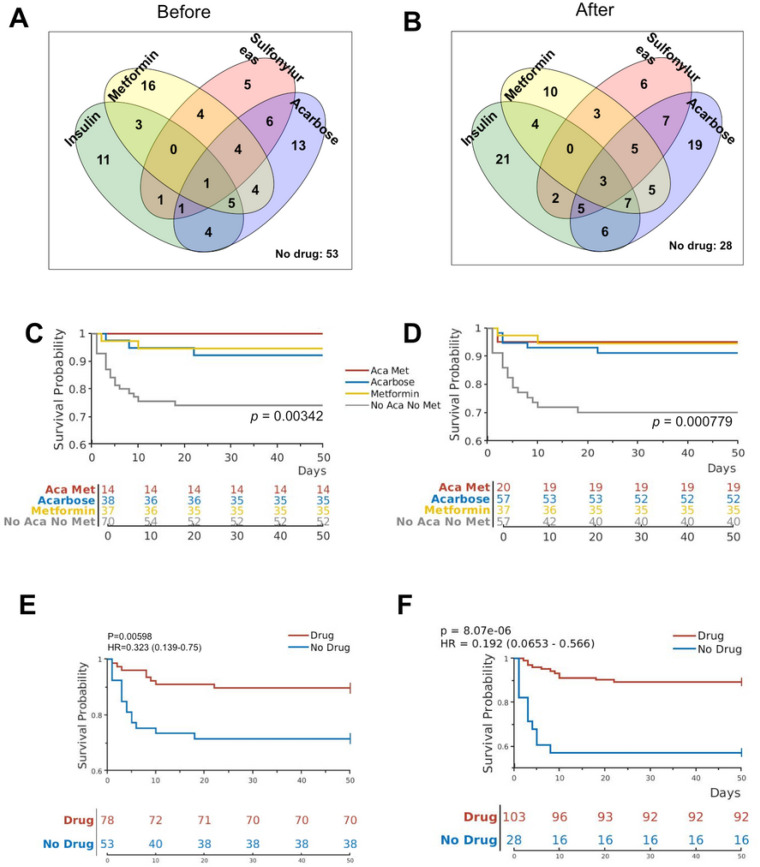
Diabetes medications and effects on survival COVID-19 patients with T2DM. (A, B) The Venn diagrams show the number of patients taking different diabetes medications before (A) and after (B) admission into the hospital. (C, D) Kaplan-Meier curves show survival probabilities for patients on different combinations of metformin and acarbose before (C) and after (D) admission. P-values indicate differences among the 4 groups by log rank statistics. (E, F) Kaplan-Meier curves show survival probabilities for patients on one or more diabetes medications vs those not on any of the four diabetes medications before (E) and after (F) admission.

We have previously reported the characteristics of a cohort of 131 COVID-19 patients with T2DM measured at admission, including age, BMI, serum glucose concentration, and oxygen saturation ^6^. These patients were managed with one or more diabetes medications, including insulin, metformin, sulfonylureas, and acarbose, or without any medications (Table 1; Fig. 1A, B). While most patients continued on the same drug(s) they had been taking, a minority of patients changed medication after admission (Fig. 1A, B).

To study the effects of diabetes medications during hospitalization on the outcome of COVID-19 patients with T2DM, we analyzed the same cohort of 131 patients using mortality as a dependent variable and medications as independent variables. When medication use after admission was analyzed for this cohort of patients, significant associations with survival were found for both metformin (p = 0.02) and acarbose use (p = 0.04), but not for insulin, and sulfonylurea (Table 1).

After admission to the hospital, patients were continued with their outpatient Insulin and oral agents.

In addition, some patients received new medications for diabetes, such as insulin, sulfonylureas, and acarbose, while the number of patients on metformin remained unchanged (Fig. 1A, B; Table 1). Among the 57 patients who used acarbose after admission, 52 survived (91.2%), and 5 (8.8%) did not survive. This finding was significantly different (p = 0.04) from those not on acarbose, with 18 (24.3%) deaths and 56 (75.7%) recoveries (Table 1), suggesting that, similar to metformin, acarbose use during hospitalization for COVID-19 was also associated with survival benefit.

### Effect of combined metformin and acarbose use on COVID-19 patients with T2DM

Since some of these patients used more than one medication, we sought to determine if using a combination of any two diabetes medications carries benefits for survival. To this end, we examined survival of this cohort of patients on two of the four diabetes medications before and after admission, comparing with those who were not on either of the two drugs. We found that patients who used both metformin and acarbose, but none of any other combinations, had significantly better chance of survival both before and after admission. Of the 131 patients of COVID-19 with diabetes, 14 were using both metformin and acarbose with or without other diabetes medications before admission. All of these 14 patients survived (100%), which was significantly different (*p* = 0.033) when compared with the 70 patients who were not on either metformin or acarbose, of whom 18 (25.7%) died and 52 (74.3%) survived (Table 2). After admission, more patients used acarbose, and a total of 20 patients used both metformin and acarbose, while 57 used neither. Of the 20 dual-use patients, one (5.0%) died and 19 (95.0%) survived, which is significantly different (p = 0.030) from the 57 who used neither [of which 17 (29.8%) died and 40 (70.2%) survived] (Table 2).

The effects of combined metformin and acarbose use on survival were further analyzed using Kaplan-Meier curves and the log-rank statistics. Those taking metformin or acarbose or both were found to have significantly better chance of survival when compared with those on neither metformin nor acarbose either before (Fig. 1C) or after admission (Fig. 1D). In addition, the COVID-19 patients with T2DM taking any one of the four medications either before and after admission had significantly better chance of survival than those who did not take any of the four medications (Fig. 1E, F).

### The effect of combined metformin and acarbose on survival is independent of other known confounders

To determine whether any of these confounding factors were disproportionately distributed in patients taking metformin or acarbose or both, thus skewing their effects, we carried out analysis of covariance for patients grouped by taking either metformin or acarbose, or both or neither. We found that there were no significant differences in the distribution of these confounding factors in different groups of patients as mentioned above, except for HbA1c (Table 3). Patients taking metformin alone or with acarbose had significantly higher levels of HbA1c than those taking neither drugs (Table 3; also see refs). However, higher levels of HbA1c were adversely associated with survival, as we have previously shown ^6^. Thus, the beneficial effects of taking both metformin with or without acarbose on survival were not attributable to the differences in HbA1c levels in these patients. In addition, significant differences were found in the distribution of patients taking or not taking sulfonylureas among these four groups of patients taking different combinations of metformin and acarbose (Table 3). In this case, the differences were due to more patients not taking any drugs (49), and an imbalance between patients taking sulfonylureas and acarbose (12) vs. those taking sulfonylureas and metformin (3), whereas the same number of sulfonylureas-taking patients (8 each) who also took both metformin and acarbose as those who took none of these two drugs (Table 3). Since patients taking sulfonylureas alone or with either acarbose or metformin were not significantly associated with better survival (Table 1, 2), we interpret that additional use of sulfonylureas does not seem to be associated with survival benefits to patients taking metformin or acarbose or both.

Other factors we have previously found significantly associated with survival of this cohort of patients were not significantly differently distributed in patients taking metformin or acarbose or both vs those taking neither. These factors include age, BMI, glucose, triglyceride, CRP D-dimer, and steroid use (Table 2, 3).

## Discussion

COVID-19 has had a major global impact and our therapeutic strategies are still limited. This retrospective study aims to evaluate existing medications for possible repurposing. Our study of a cohort of COVID-19 patients with T2DM indicates that use of metformin and acarbose together both prior to hospitalization and during treatment in hospital is significantly associated with lower mortality in COVID-19 patients with T2DM. This finding should provide a basis for designing randomized, controlled clinical trials testing the effects of use of metformin or acarbose or both on COVID-19 patients with or without T2DM.

COVID-19 has spread globally with heavy impact on most countries, yet despite many ongoing and concluded clinical trials, we are still limited in therapies. The anti-viral drug remdesivir is perhaps the most promising agent, yet it has shown rather modest benefits ^1, 16^. Thus, there is need for alternative or adjunctive therapies to combat COVID-19. There have been trials of hydroxychloroquine with or without azithromycin ^17, 18^ and tocilizumab ^19^, an antibody that inhibits interleukin-6 receptor ^20–22^ and angiotensin converting enzyme inhibitors and angiotensin receptor blockers ^23^, to name a few, all with modest if any outcome benefits to date.

We have recently reported that metformin use prior to hospital admission is associated with reduced mortality for COVID-19 patients with diabetes ^6^, suggesting metformin as a potential COVID-19 therapeutic. We have previously shown for this cohort of 131 patients that age, body weight, BMI, oxygen desaturation, glucose, triglyceride, C-reactive protein (CRP), D-dimer levels, and steroid use were significantly associated with mortality ^6^, consistent with reports of other cohorts of COVID-19 patients ^3^. Metformin is a widely available safe and inexpensive drug that has been suggested for use in various clinical settings other than diabetes ^24^. This study has found that acarbose, used alone or together with metformin during treatment, is associated with reduced mortality of COVID-19 patients with T2DM. Acarbose is another inexpensive drug that is widely used at least in China. Since metformin or acarbose alone is beneficial, taking both might have additive or even synergistic effects. Whether the combination of metformin and acarbose is effective treatment should await future randomized trials. These analyses suggest that use of metformin or acarbose or both is associated with improved survival of COVID-19 patients with T2DM independent of their effects on lowering glucose concentration and body weight.

As with all retrospective studies, the current study has limitations. First, the sample size is modest and not large enough for propensity score matching in covariate analysis. Although we have controlled for known confounders in the data analyses, there may still be residual confounding. However, given the magnitude of the survival differences we have observed, we doubt some unrecognized covariate would have such a major impact. Nonetheless we welcome further data to confirm or refute our findings. Second, T2DM patients taking metformin or acarbose or both might have different disease severity or vulnerability than those under other management. Nonetheless, these new observations may be important and could be a basis for a more definitive randomized trial regarding pharmacotherapy for inpatient DM in COVID-19.

## Conclusions

The optimal therapy for inpatients with DM and COVID-19 is unclear. Our findings support the investigation of oral agents for glucose control in this context given a potential impact on clinical outcomes.

## Figures and Tables

**Table 1.** Medication Use for COVID-19 Patients with Type-2 Diabetes after admission

		All	Recovered	Death	*p*-value
Total, n(%)		131	108 (82.4)	23 (17.6)	
Insulin	Y	48	40 (83.3)	8 (16.7)	0.999
	N	83	68 (81.9)	15 (18.1)	
Metformin, n(%)	Y	37	35 (94.6)	2 (5.4)	0.0222*
	N	94	73 (77.7)	21 (22.3)	
Sulfonylureas n(%)	Y	31	29 (93.5)	2 (6.5)	0.103
	N	100	79 (79.0)	21 (21.0)	
Acarbose, n(%)	Y	57	52 (91.2)	5 (8.8)	0.0376*
	N	74	56 (75.7)	18 (24.3)	
At least one above drug		103	92 (89.3)	11 (10.7)	2.85e-4***
None of above drugs		28	16 (57.1)	12 (42.6)	

Y: used the indicated medication. N: did not used the indicated medication. Fisher’s exact test was used to calculate *p*-values for difference in survival between Y and N groups.

**Table 2.** In-hospital use of two medications in COVID-19 Patients with Type-2 Diabetes

		Survival	Death	*p*-value
Total, n(%)	131	108 (82.4)	23 (17.6)	
+Metformin +Insulin	14	13 (92.9)	1 (7.1)	0.2749
−Metformin −Insulin	60	46 (76.7)	14 (23.3)	
+Metformin +Sulfonylureas	11	10 (90.9)	1 (9.1)	0.2787
−Metformin −Sulfonylureas	74	54 (73.0)	20 (27.0)	
+Metformin +Acarbose	20	19 (95.0)	1 (5.0)	0.0304*
−Metformin −Acarbose	57	40 (70.2)	17 (29.8)	
+Insulin +Sulfonylureas	10	9 (90.0)	1 (10.0)	0.6764
−Insulin −Sulfonylureas	62	48 (77.4)	14 (22.6)	
+Insulin +Acarbose	21	19 (90.5)	2 (9.5)	0.1971
−Insulin −Acarbose	47	35 (74.5)	12 (25.5)	
+Acarbose +Sulfonylureas	20	18 (90.0)	2 (10.0)	0.1339
−Acarbose −Sulfonylureas	63	45 (71.4)	18 (28.6)	

Patients using two indicated medications were compared with those not using the two indicated medications (patients using only one of the two medications were not included). Fisher’s exact test was used to calculate *p*-values for difference in survival.

**Table 3.** Patients with and without both acarbose and metformin after admission

		All	+Aca+Met	+Aca−Met	−Aca+Met	−Aca−Met	*p*-value	
**Survival**								
Survived, n(%)		108 (82.4)	19 (95.0)	33 (89.2)	16 (94.1)	40 (70.2)	0.0304	*
Death, n(%)		23 (17.6)	1 (5.0)	4 (10.8)	1 (5.9)	17 (29.8)		
**Characteristics and Medication**								
Gender								
Male, n(%)		74 (56.5)	12 (60.0)	25 (67.6)	10 (58.8)	27 (47.4)	0.437	
Female, n(%)		57 (43.5)	8 (40.0)	12 (32.4)	7 (41.2)	30 (52.6)		
Age (mean±std)		66.8±11.6	63.6±11.3	67.9±12.1	65.6±11.4	67.2±11.4	0.111	
Weight (Kg, mean±std)		66.0±10.1	67.8±17.1	67.2±8.45	63.9±6.15	65.1±8.7	0.806	
BMI (mean±std)		24.18±3.33	24.46±4.70	24.21±3.33	24.08±1.91	24.21±3.22	0.999	
O2Saturation (mean±std)		0.91±0.13	0.92±0.10	0.92±0.10	0.94±0.04	0.88±0.16	0.279	
Cholesterol (mmol/L, mean±std)		3.84±1.09	4.01±0.80	3.60±0.76	4.04±0.92	3.88±1.42	0.34	
Triglyceride (mmol/L, mean±std)		1.32±0.76	1.53±0.98	1.30±0.52	1.25±0.52	1.27±0.89	0.259	
HDL (mmol/L, mean±std)		0.95±0.34	1.03±0.34	0.89±0.29	1.00±0.33	0.95±0.37	0.428	
LDL (mmol/L, mean±std)		2.41±0.85	2.44±0.77	2.29±0.57	2.59±0.89	2.42±1.04	0.688	
HbA1c (mmol/L, mean±std)		7.89±1.85	9.70±1.99	7.82±1.56	8.13±2.95	7.26±1.45	0.0092	**
Glucose (mmol/L, mean±std)		9.03±4.45	10.43±5.27	9.17±4.65	7.97±3.96	8.77±4.13	0.183	
CRP (mg/L, mean±std)		49.37±65.45	73.69±93.56	47.24±56.26	40.94±45.11	44.80±64.74	0.404	
D-dimer (mg/L, mean±std)		4.27±12.16	3.54±7.76	7.85±20.35	0.87±0.82	3.03±4.99	0.218	
CAD, n (%)	Y	28 (21.4)	6 (4.6)	6 (4.6)	1 (0.8)	15 (11.5)	0.775	
	N	103 (78.6)	14 (10.7)	31 (23.7)	16 (12.2)	42 (32.1)		
Hypertension, n(%)	Y	78 (59.5)	13 (9.9)	22 (16.8)	10 (7.6)	33 (25.2)	0.609	
	N	53 (40.5)	7 (5.3)	15 (11.5)	7 (5.3)	24 (18.3)		
Hypert’n Med, n(%)	Y	45 (34.4)	9 (6.9)	17 (13.0)	6 (4.6)	13 (9.9)	0.0841	
	N	86 (65.6)	11 (8.4)	20 (15.3)	11 (8.4)	44 (33.6)		
Hyperlipidemia, n(%)	Y	14 (10.7)	2 (1.5)	6 (4.6)	1 (0.8)	5 (3.8)	0.999	
	N	117 (89.3)	18 (13.7)	31 (23.7)	16 (12.2)	52 (39.7)		
Insulin, n(%)	Y	35 (26.7)	6 (4.6)	11 (8.4)	4 (3.1)	14 (10.7)	0.768	
	N	96 (73.3)	14 (10.7)	26 (19.8)	13 (9.9)	43 (32.8)		
Sulfonylureas n(%)	Y	31 (23.7)	8 (6.1)	12 (9.2)	3 (2.3)	8 (6.1)	0.0233	*
	N	100 (76.3)	12 (9.2)	25 (19.1)	14 (10.7)	49 (37.4)		
Steroid Use, n(%)	Y	48 (36.6)	4 (3.1)	14 (10.7)	4 (3.1)	26 (19.8)	0.0619	
	N	83 (63.4)	16 (12.2)	23 (17.6)	13 (9.9)	31 (23.7)		
**Oxygen Therapy and Ventilation, n(%)**								
Room Air		26 (19.8)	2 (1.5)	7 (5.3)	4 (3.1)	13 (9.9)	0.212	
Nasal cannula		69 (52.7)	12 (9.2)	21 (16.0)	11 (8.4)	23 (17.6)		
Non-rebreather mask		7 (5.3)	0 (0.0)	2 (1.5)	0 (0.0)	5 (3.8)		
High Flow		7 (5.3)	1 (0.8)	1 (0.8)	0 (0.0)	5 (3.8)		
Non-invasive ventilation		19 (4.5)	2 (1.5)	5 (3.8)	2 (1.5)	10 (7.6)		
Invasive Ventilation		3 (2.3)	1 (0.8)	1 (0.8)	0 (0.0)	1 (0.8)		

Measurements were from patient’s blood samples obtained at admission. p-values indicate the level of significance in the distribution of each independent variable (e.g., Insulin Y and N) among the 4 groups of metformin acarbose use combinations, as calculated by Chi-square test, or in case of continuous independent variable (e.g., CRP), by logical regression analysis.
